# Ephemeral Emotional Resonance: User-Perceived Functional Value Leading to Short-Form Video Use

**DOI:** 10.3390/bs15030341

**Published:** 2025-03-10

**Authors:** Xinzhou Xie, Yanjun Lin, Qiyu Bai

**Affiliations:** 1 School of New Media, Peking University, Beijing 100871, China; xzxie@pku.edu.cn; 2 State Key Laboratory of Media Convergence and Communication, Communication University of China, Beijing 100024, China; linyj@cuc.edu.cn

**Keywords:** short-form video, perceived functional value, bridging social capital, internet self-efficacy, moderated mediation model

## Abstract

With the rapid development of short-form videos, more and more people have gained a deep understanding of the functional value of short-form videos. Based on structuration theory, this paper explores the reasons why users use short-form videos. The authors conducted a questionnaire survey on 2613 Chinese short video users, and found that bridging social capital played a mediating role between users’ perceived functional value of short-form videos and their use of short-form videos. In addition, internet self-efficacy played a moderating role. This finding not only enhances our understanding of the value of short-form videos and users’ relational needs, but also highlights the crucial role of self-efficacy in users’ engagement with this new medium.

## 1. Introduction

Short-form video platforms, such as TikTok, YouTube Shorts, and Instagram Reels, have rapidly gained global popularity and attracted a demographically diverse audience. With videos typically ranging from 30 to 60 s and an auto-scrolling feature, these platforms are designed to engage users quickly and seamlessly, fitting the fast pace of contemporary life (Y. Wang, 2020; David & Roberts, 2024). This design not only enhances user interactivity but also reinforces mobilization capacity, making short-form videos increasingly significant in fields like subculture communication (Kaur-Gill, 2023), political discourse (Janis et al., 2024), health communication (Oh et al., 2024), and marketing (Barta et al., 2023).

However, as versatile and gamified social media platforms, short-form video services present notable divergence in how users experience agency. On the one hand, the addictive design and algorithm-driven content recommendations of short-form videos have raised concerns. Several studies highlight that users, influenced by continuous information streams, may develop media addiction, which could undermine their sense of agency and self-determination (De Bérail et al., 2019; Rach & Peter, 2021; Ye et al., 2022). On the other hand, many digital media analysts argue that these platforms also foster user agency, as they allow individuals to shift from passive recipients to active participants. The easy-to-use features of these platforms, such as background music and lip-syncing templates, enable users to personalize their engagement, interact with content, and participate in social discourse (Montag et al., 2021; Menon, 2022; Qin et al., 2022). Given the pervasive influence of short-form videos on public life, understanding how users perceive platform functionalities and what motivates their engagement is essential for assessing their broader societal impact.

One crucial aspect of user engagement on short-form video platforms is their potential to facilitate the bridging of social capital, which serves as a key motivation for active participation. Bridging social capital refers to resources generated through external, weak ties (M. S. Granovetter, 1977). Research suggests that short-form video platforms, compared to other social media platforms, are particularly effective at fostering such connections (Lai, 2022). For example, TikTok’s algorithm-driven recommendation system amplifies content visibility beyond local and close-knit connections, ensuring global reach upon upload (Vassey et al., 2025). This expanded exposure helps users satisfy their desire to build bridging social capital. While previous studies on short-form videos have not distinctly separated the motivation to form new social ties from the motivation to maintain close relationships (Omar & Dequan, 2020), the role of bridging social capital in this context remains a critical area of interest.

Therefore, this study aims to emphasize user agency in short-form video use, specifically examining whether bridging social capital mediates the relationship between perceived functional value and short-form video use. Next, we examine whether internet self-efficacy serves as a moderator in this mediation effect. The data analysis is then presented, followed by a discussion of the findings, limitations, and potential directions for future research.

## 2. Theoretical Background and Hypothesis Development

### 2.1. Our General Theoretical Framework: Giddens’ Structuration

At a foundational theoretical level, this study is grounded in Anthony Giddens’ structuration theory (Giddens, 1986), which provides a comprehensive framework for understanding the dynamic, bidirectional relationship between individual social agents (micro-level) and social structures (macro-level). Specifically, social structures—such as institutions, norms, and technological frameworks—both enable and constrain individuals’ daily routines. At the same time, individuals actively shape and transform these structures, a process known as the ‘duality of structure’. This theory emphasizes reflexive monitoring, meaning that individuals continuously assess and adjust their actions based on the structural conditions they encounter and the needs they have (Giddens, 1984).

Following structuration theory, many digital media analysts have explored the relationship between individual users and technological structures and systems and found ‘agency’ to be invaluable in users’ media participation (Van Dijck, 2009; Sujon et al., 2018; Schnauber & Mangold, 2020). For example, user-generated content is regarded as one of the most important ‘agency’ criteria for being active producers rather than passive consumers in the initial stage of Web 2.0. With the evolving functions of social media, it is essential to rate different levels of participation for ‘user agency’ (Van Dijck, 2009). As a new form of social media, short-form video platforms indeed expand ‘user agency’. It is therefore essential to examine how short-form video platform affordances influence user engagement while simultaneously being shaped by user agency, and ultimately foster user engagement.

### 2.2. Perceived Functional Value and Bridging Social Capital

Perceived functional value is one aspect of this ‘perceived value’, referring to the subjective utility perception of the user (Zeithaml, 1988; Teas & Agarwal, 2000): When users are interested in a new product, they will subjectively evaluate the overall profit/loss before they make their final purchase/use decision (Woodruff, 1997). In the social media context, if the platform is equipped with a high level of media features and a high level of technology affordance, users will manifest more perceived functional value (Sheer, 2011).

Media scholars are prone to relate media features to social interaction. One of the most common ways to measure social interaction is bridging social capital. It refers to cross-cutting social relationships and resources (Paxton, 1999). This kind of interaction occurs in an alien environment when two different groups encounter one another, either by chance or intentionally, with the need to seek support or exchange information (Larsen et al., 2004). This comes under the notion of ‘weak ties’ (M. Granovetter, 1983), that is to say, it is used mostly for random reciprocal relationships, with these relationships being neither fixed nor enduring (Putnam, 2001). In this paper, we define bridging social capital as the intention of users to make the acquaintance of people outside their real lives and to try to achieve a more fluid class system. Examples would involve communicating with celebrities in online communities.

Structuration theory (Giddens, 1986) suggests that individuals maintain a degree of agency when interacting with social structures. Actors can reflect on the daily routine before entering the next round of social practice. When human objectives are not being achieved, actors will consider a new idea to participate in social interaction (Giddens, 1986). This concept of agency aligns with the active role that users play in selecting media to fulfill specific needs, which is further elaborated by the micro-level framework of Uses and Gratifications Theory (UGT), examining how individuals actively select media based on their personal and social needs (Katz et al., 1973).

Past research has applied UGT to examine how users continuously adapt their communication patterns in response to emerging social media features (Schrock, 2015). This adaptation is particularly evident on short-form video platforms, where users exercise their agency by selecting and utilizing interactive features that align with their needs for social connection. For example, young users engage with duets and remixes, which allow them to respond to or collaborate with others, reinforcing a sense of connection. Additionally, users actively leverage platform-generated memes as a form of creative expression, cultivating a ‘collective experience’ through the viral spread and sharing of videos (Zulli & Zulli, 2022). A notable example of this dynamic occurred during the 2019/20 Australian bushfires. As Brown et al. (2024) observe, TikTok users employed memes—such as juxtaposition, whimsy, and humor—to express emotions like anger and frustration, thereby deepening emotional engagement and fostering solidarity within online communities.

Short-form video platforms, therefore, are no longer mere devices for providing videos for on-demand service, but they also offer everyone a chance to become short-form video content creators and even enable users to connect with the wider world. In other words, users of short-form video platforms perceive the functional value they need subjectively—such as bridging social capital—and this may stimulate user perception concerning expanding social networks. Consequently, short-form video users will perceive short-form video platforms as a tool by which to establish and maintain bridging social capital. Therefore, the following hypothesis is tested in this study:

**Hypothesis** **1:** *The perceived functional value of a short-form video platform relates positively to user motivation concerning bridging social capital on platforms.*

### 2.3. Bridging Social Capital and Short-Form Video Use

According to the theory of structuration, resources play a crucial role in shaping individuals’ decisions to take action. Among these resources, social capital serves as an important facilitator for social interactions, as it enables individuals to access information, opportunities, and support networks. Humans inherently have a basic security system, generating the need to ensure some degree of predictability for social life (Giddens, 1984). In this context, social capital provides a sense of security by fostering connections that help individuals integrate into broader social systems. It follows, therefore, that people’s social needs influence their engagement with digital platforms, where they seek interaction, information, and social belonging.

Earlier studies have also suggested a predictive effect of bridging social capital on social media use. Boyd (2008) suggests that socializing with peers is one of the most important motivations behind teenagers’ usage of the SNS. Shane-Simpson et al. (2018) compare Facebook, Instagram, and Twitter, arguing that Twitter tends to attract users who seek to gain bridging social capital due to its relatively lax use of privacy settings. A quantitative study further confirmed this relationship, showing that a high degree of bridging social capital significantly influences user satisfaction and continued social media use (Chang & Zhu, 2012). Yoon (2014) explains this phenomenon by arguing that bridging social capital reduces information uncertainty, leading users with high bridging social capital to engage more frequently with SNS platforms to expand relationships and access new information.

Given that social capital is a critical driver of online interactions, understanding whether or not this motivation will affect users’ engagement with short-form video platforms, which represent a new form of social media, is essential. UGT offers a useful framework for examining these motivations, as it explains why audiences engage with different types of media to fulfill their social needs (Katz et al., 1973). On short-form video platforms, social gratification is enhanced by three key features. First, the auto-scrolling mechanism ensures that users’ uploaded videos are automatically distributed to wider audiences, increasing the chances of content being seen and expanding the users’ social reach. Second, users are able to display their bodies through the use of the televisual medium. As such, users’ needs for self-representation are likely to increase. This, in turn, increases the demand for expanded connectivity (Przybylski et al., 2013). Third, users can actively leverage TikTok’s deep learning-driven systems and algorithmic recommendations to explore content beyond their immediate social circles (Liu et al., 2022; Gerbaudo, 2024). By broadening their content exposure, users create more opportunities to form weak ties and expand their bridging social capital.

As users actively seek new social connections and engagement through these platform features, their desire to expand their social networks and gain new information becomes a key driver of increased short-form video platform use. We therefore propose the following hypothesis to examine the relationship between bridging social capital and short-form video use:

**Hypothesis** **2:** *Bridging social capital relates positively to short-form video use.*

### 2.4. Perceived Functional Value, Bridging Social Capital, and Short-Form Video Use

According to the theory of structuration, the acceptance of technology and SNS use are based on a trade-off between structure and human needs; this is the case especially in terms of utility and communication apprehension (Subrahmanyam et al., 2006). Previous studies have examined how perceived value drives action in terms of customers’ SNS use or continued use (Zhang et al., 2017). As we have already hypothesized above, perceived functional value corresponded positively with short-form video use and the motivation for obtaining bridging social capital was related to short-form video use. Indeed, with a large number of external functions and a high degree of subjective intention, users are likely to generate more perceptions of social value and, consequently, be stimulated into more short-form video use. This is consistent with UGT, which highlights that users actively engage with media to satisfy their needs. Thus, bridging social capital might well have a substantial mediation effect on behavioral intention in terms of engagement in short-form media use. Therefore, all this leads us to state and test the following hypothesis:

**Hypothesis** **3:** *Bridging social capital mediates the relationship between perceived functional value and short-form video use.*

### 2.5. Internet Self-Efficacy

Internet self-efficacy is a widely used construct. This construct indexes the confidence of users in their ability to adopt new technology or new internet services (Daugherty et al., 2005). Users with strengthened beliefs in their own abilities are more likely to master a new product (Ajzen & Sexton, 1999). This metacognitive ability to reflect on oneself and evaluate the effectiveness of one’s thoughts and actions represents the most fundamental and uniquely human aspect of agency (Bandura, 2006). Research has found that internet self-efficacy can play a moderating role between social media cognitions and social media motivations (Fan et al., 2019; Kim & Um, 2016). Individuals with higher internet self-efficacy are typically more confident in engaging in social interactions on social platforms. In contrast, individuals with lower internet self-efficacy may experience more anxiety due to uncertainty about their personal abilities in using the internet, which can reduce their interactions on social networks (J. L. Wang et al., 2015; Jokisch et al., 2020).

This is particularly relevant on short-form video platforms, where the ease of creating, editing, and sharing content encourages user interaction, yet requires some level of technical confidence. Features such as vertical video formats, built-in editing tools, and algorithmically curated recommendations lower the barrier to entry, making it easier for users to participate and create (Liang, 2022). However, users with lower internet self-efficacy may still struggle to take full advantage of these features, thereby limiting their engagement. During the construction of social capital, individuals with high internet self-efficacy can fully comprehend the interaction features of short-form video platforms, such as user-generated content and viral trends, enabling them to quickly create weak ties through shared content like memes or challenges. Consequently, individuals with high internet self-efficacy tend to manifest a positive correlation between perceived functional value and bridging social capital. This, then, leads to our fourth and final hypothesis:

**Hypothesis** **4:** *Internet self-efficacy moderates the relationship between perceived functional value and bridging social capital, so that the positive relationship between perceived functional value and bridging capital is stronger for individuals with high internet self-efficacy.*

**Hypothesis** **5:** *Internet self-efficacy moderates the indirect effect of perceived functional value on short-form video use via bridging social capital, so that the indirect effect is stronger for individuals with higher levels of internet efficacy.*

**Figure 1:**
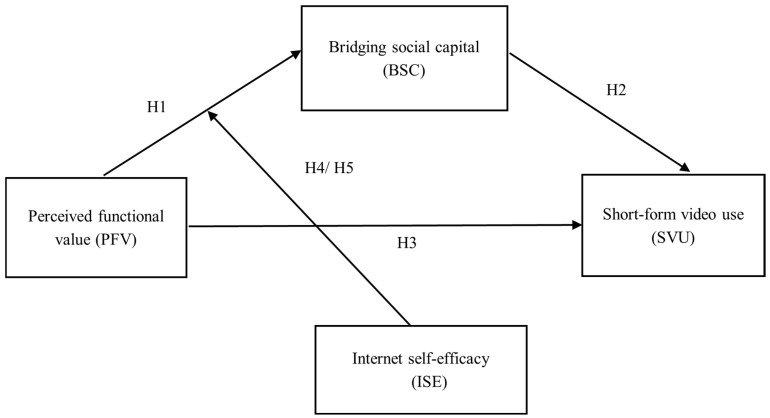
Proposed research model with hypotheses.

We summarize the assumptions in a model diagram; see Figure 1 for details.

## 3. Research Method

### 3.1. Data Collection

Data collection was conducted by means of a computerized self-report questionnaire survey of Chinese netizens carried out by IPSOS (China), a professional institute dedicated to market research. All netizen short-form video user respondents responded anonymously. The sample for this study included 3000 internet users, with sampling made in accordance with Chinese netizens’ structure reported by CNNIC 45 (CNNIC, 2020). Next, volunteers who chose the ‘never use short video’ option in the ‘jump options’ section were excluded. As a result, the final sample size was 2613. The average age was 32.25 years (SD = 11.00). In the database, 51.2% of the participants were male and 48.8% were female. A total of 15.7% of participants were from a first-tier Chinese city, 35.2% were from a second-tier one, and 49.1% were from a third-tier one. In terms of educational background, 8.7% had attended a junior high school and below, 49.1% a senior high school and below, 21.9% a junior college, and 20.3% a college and above. All the participants were guaranteed anonymity, and informed consent was obtained before commencing the online survey. The study was guaranteed in terms of confidentiality and compliance.

### 3.2. Variable

Short-form video use. To measure the use of short-form videos, we included six items to indicate how often and how much users used a short-form video application. For example, “I spend some time on watching short-form videos every day”. These items were assessed on a five-point scale, ranging from “strongly disagree” to “strongly agree” (Cronbach α = 0.736).

Short-form video perceived functional value. Short-form video perceived functional value originated from the perceived value scale (Sweeney & Soutar, 2001) modified by (Rintamäki et al., 2006), suggesting that perceived functional value is instrumental. It consists of saving money and convenience. In the context of short-form video, we focus on four aspects from the convenience dimension: access, search, possession, and transaction. These comprised seven items, for example, “By watching short-form videos, I can learn useful information”, which was rated by participants on a five-point scale (1 = strongly disagree and 5 = strongly agree). For the current study, the scale showed feasible reliability (Cronbach α = 0.733).

Bridging social capital. The measures used for the motivation of bridging social capital were adapted from Williams (2006). The scale consists of three items, for example, “Engaging with short-form video brings me new friends”, again on a five-point scale, ranging from “strongly disagree” to “strongly agree” (Cronbach α = 0.737).

Moderator and covariates. The moderator variable that we chose to examine was internet self-efficacy, which was assessed by averaging five adapted items from Zheng (2012), for example “I am good at searching for information on internet”. The variables of participants’ gender, age, and educational level were controlled, as former studies showed that they might affect social capital and short-form video use (Cronbach α = 0.728).

### 3.3. Statistical Analysis

Firstly, descriptive statistics and correlation matrixes were calculated. Secondly, the PROCESS macro (Model 4), developed by Hayes in 2012 (Hayes, 2012), was used to test the mediation effect of bridging social capital between perceived functional value and short-form video use. Thirdly, the PROCESS macro (Model 7) was used to examine whether educational background moderated these mediation processes. In addition, this study employed the bootstrapping method (Hayes & Scharkow, 2013), which processed 95% bias-corrected confidence intervals from 5000 samples of data in order to ensure the significance of indirect effects. The results indicated that these effects were significant when the confidence intervals excluded zero.

## 4. Results

The main aims of this study were as follows: firstly, we aimed to explore whether bridging social capital mediates the relationship between perceived functional value and short-form video use. Next, we aimed to explore whether internet self-efficacy is the moderator during this mediation effect. The analysis followed three steps.

### 4.1. Preliminary Analyses

Descriptive statistics (means, standard deviations, and zero-order correlations) for all the variables are presented in Table 1 below. The internal consistency alphas were all above 0.70. The results showed that perceived functional value was positively correlated with bridging social capital and the use of short-form video. Moreover, bridging social capital was positively associated with short-form video use. Lastly, users’ internet self-efficacy is positively related to the perceived functional value of short-form video, bridging social capital, and their short-form video use.

### 4.2. Testing for a Mediation Effect

Table 2 shows the results relating to Hypotheses 1, 2, and 3. The results revealed that perceived functional value positively related to bridging social capital (B = 0.630, SE = 0.027, *p* < 0.001; see Model 1 of Table 2), and bridging social capital was positively associated with short-form video use (B = 0.326, SE = 0.012, *p* < 0.001; see Model 2 of Table 2). Hence, Hypotheses 1 and 2 were supported.

Finally, it was found that perceived functional value had an indirect effect on short-form video use (B = 0.206). Bootstrapping results confirmed the significance of the indirect effect, with a 95% confident interval of [0.182, 0.230]. Therefore, Hypothesis 3 was also supported.

### 4.3. Testing for Moderation Effect

The results relating to Hypothesis 4 are reported in Table 3. The results demonstrate that the interaction of perceived functional value and internet self-efficacy significantly predicts bridging social capital (B = 0.207, *p* < 0.001. see Table 3), supporting Hypothesis 4.

**Figure 2:**
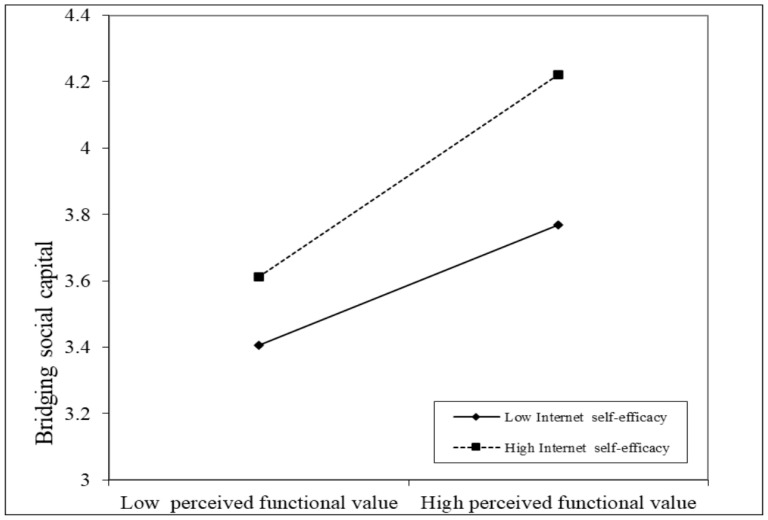
The interaction effect of perceived functional value and internet self-efficacy on bridging social capital. High and low levels of perceived functional value and internet self-efficacy represent one standard deviation above and below the mean, respectively.

We then plotted a slope that illustrated the relationship between the perceived functional value of short-form video and bridging social capital for the low and high levels of internet self-efficacy, respectively. As Figure 2 shows, the slope of the relationship between the perceived functional value and bridging social capital with high internet self-efficacy was strong (B_high internet self-efficacy_ = 0.595, t = 18.426, *p* < 0.001), while the slope with low internet self-efficacy was relatively weak (B_low internet self-efficacy_ = 0.354, t = 11.034, *p* < 0.001). Thus, Hypothesis 4 was supported.

Next, the indirect effects of the perceived functional value on short video use through bridging social capital were tested. The index of moderated mediation was significant (B = 0.068, SE = 0.017, 95% CI = [0.039, 0.104]). We also examined the conditional indirect effects of perceived functional value on short-form video use via bridging social capital at varying levels of internet self-efficacy (1 SD above the mean and 1 SD below the mean) using Bauer et al.’s (2006) method. For participants with a higher level of internet self-efficacy, perceived functional value had a more indirect effect on motivation for bridging social capital (B = 0.194, SE = 0.017, 95% CI = [0.163, 0.229]) compared with those with low internet self-efficacy (B = 0.113, SE = 0.018, 95% CI = [0.077, 0.146]). Hypothesis 5 was therefore supported.

**Figure 3:**
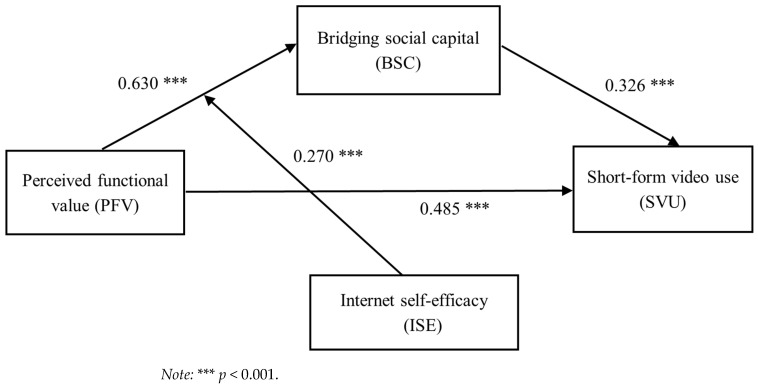
Summary results of moderated mediation analysis.

Summary results of moderated mediation analysis are presented in Figure 3 below.

## 5. Discussion and Conclusions

This study illuminates and analytically traces the relationship between perceived functional value and engagement with short-form video. In carrying this out, it also proposes a promising theoretical framework that adapts Giddens’ structuration theory by grafting into it the constructs of perceived value (together with the constructs of bridging social capital and internet self-efficacy). In this way, our research can best contribute to the potential role of human agency in maximizing human benefit and minimizing the problematic effects of social media’s effects on individuals and society as a whole.

More specifically, our study highlights how perceived functional value emerges with and adapts to users’ social needs in a short-form video context—needs that can increasingly be referred to as ‘equal social interaction’. Additionally, and in line with Giddens’s notion of the ‘cycle of structuration’ (Giddens, 1986), our study elucidates how this phenomenon may, in fact, be feeding back into the technology, and, if not exactly modifying the technological structure itself (short-form video platforms), then at least altering its functional position in the total social structure. Therefore, exploring how perceived functional value creates bridging social capital and how it can contribute to the betterment of short-form video’s role in society is an extremely meaningful and worthwhile endeavor.

Our results showed that users’ perception of functional value and their intentionality of building bridging social capital increased short-form video use. This is consistent with some previous studies, which argue that social media’s technological characteristics are beneficial in users’ establishing new relationships (Ellison et al., 2007). Additionally, the relationship between perceived functional value and bridging social capital was strong in the case of high internet self-efficacy.

At face value, the current study extends the understanding of users’ motivation for social media, as proposed by Haridakis and Hanson (2009). In that previous study, enhancing close relationships can be seen as the main motivation for social media users (Stern & Taylor, 2020). Yet, in contrast, our study empirically suggests that short-form video’s technical affordance, such as, provided video or audio memes, is distinct from that of such earlier-established social networking sites. With these functionalities, users may spend less effort on self-presentation and social identity in online communications and utilize short-form videos to establish weak-tie relationships. These relationships may be intentionally and deliberately formed by the users themselves to be equal, rather than hierarchical as before. For example, when Chinese National Day approaches, many Chinese users may typically use the Chinese patriotic song “My Motherland and Me” as background music and sing along with the music to express their patriotic emotions. This piece of short-form video content alone may well automatically strengthen weak ties between these disparate users within a short time without any need for users to supply additional and specially created content (e.g., additional text or photos). In other words, users’ means of managing social interaction and social relationships have been enriched in short-form video use.

This transition, from deeper, more substantial, and complex modes of social interaction through social media engagement to easier-to-use and more interactionally shallow forms, can perhaps be best understood with reference to the best-selling yet academically influential book “Alone Together” by Sherry Turkle (Turkle, 2011). Turkle argues that communication technology often gives us the mere illusion that we have autonomy to control the pace of, and effort spent on, social life, while in fact—and on the contrary—users often begin to feel more alienated from each other, from their own social needs, and from their own social abilities—more than they ever felt before.

Furthermore, advanced social media technology may well be generating the feeling of ‘fear of missing out’ in an expanding landscape of social belonging. It seems, too, that users of advanced social media are currently stuck in a dilemma between, on the one hand, the need to achieve and maintain online autonomy and, on the other hand, the need for social belonging. Indeed, this dilemma is exacerbated at a time when social media-mediated social interactions/interpersonal relationships have just recently transitioned from modes that are more highly charged with emotional investment to ‘cooler’ and more practical modes, marked by emotional distance—rather than by closeness (Sujon et al., 2018).

Until a less dilemmatic, more humanly and emotionally appropriate—and more workable—mode of social media engagement emerges, it seems likely that short-form video engagement will continue to function as a kind of ‘cheap’ psychological compensation, which enjoys popularity among users when these users are seeking emotional connections yet with little emotional investment: the compensation of being able to feel socially connected (albeit in a merely superficial or illusory way), while at the same time being able to invest only minimal amounts of emotional energy.

This finding is of great significance to the study of political participation on short-form video platforms. Previous studies mainly focused on the unique algorithmic distribution and innovative expression features of short video platforms, which encourage more people to engage in politics through ludic engagement, enriching the perspective of individual narratives in political activities (Kennedy, 2020; Cervi et al., 2021). Some studies also pointed out that political participation in short-form videos is essentially the mobilization of emotions, where individuals align with those who share similar ideologies, forming a collective identity that resonates and strengthens common viewpoints or develops hatred toward political opponents (McLaughlin et al., 2024; Weimann & Masri, 2023). However, these studies overlook the importance of human demand for technology and the role of interpersonal relationships in human decision-making. This study, through quantitative research methods, demonstrates that it is the change in users’ perceived functionality that leads to the establishment of weak ties, which ultimately results in political participation and emotional influence on opinion markets.

Given the existing potential for easy social interactions facilitated by short-form videos, it is likely that such platforms will continue to evolve as powerful tools for political engagement. It is predicted that short-form video platforms will further enhance their ability to foster emotional connections and collective identities by refining their easy-to-use functionalities for social interactions.

In addition, governments will either be able to follow audio–visual models or create viral short-form videos, delivering an emotional connection and the ideal of civic participation. As such, governments can access certain groups of users to strengthen civic consciousness and social cohesion as well as establish ideas of democracy.

Although short-form video platforms have potential for fostering emotional connection and civic participation, their inherent focus on humor and entertainment features could, at times, lead to message discounting and overshadow more substantive content. For example, research shows that TikTok users primarily motivated by informational content may react negatively when encountering humor, often rejecting or dismissing the underlying message being conveyed (Oh et al., 2024). This suggests a need for future research to explore the relationship between user motivations, social connections, and the impact of platform functionalities on content quality and user engagement. Understanding these dynamics could help mitigate the risk of short-form videos prioritizing entertainment over valuable information and offer insights into creating a more balanced user experience.

## 6. Limitations

There are some limitations to this study. Firstly, as our data consist solely of Chinese users, the geographical limitation may affect the generalizability of the findings. Short-form video platforms are a global phenomenon, and users’ motivations, behavioral patterns, and platform usage may vary across different cultural contexts. Therefore, future research could expand data sources to include users from different countries and regions or conduct cross-cultural comparative analyses with existing studies to examine the applicability and universality of the findings. The second limitation concerns the fact that short-form video is not an exclusive function of short-form video apps; rather, it has become instrumental in other SNS apps, too. For example, Facebook and Twitter are both equipped with short-form video technology. It would, therefore, be indispensable for future studies to examine different categories of function as well as focus on specific functions like socializing, networking, and social navigation (Thelwall, 2009).

## Figures and Tables

**Table 1:** Descriptive statistics, alpha coefficients, and correlations.

	M	SD	1	2	3	4	5	6	7
1. Age	32.250	11.000							
2. Gender	1.490	0.500	−0.157 **						
3. Education	3.550	0.982	−0.220 **	0.208 **					
4. PFV	3.923	0.511	0.013	0.111 **	0.141 **	(0.733)			
5. BSC	3.506	0.767	0.095 **	−0.039 *	−0.062 **	0.402 **	(0.737)		
6. SVU	3.777	0.593	0.037	0.073 **	0.050 *	0.594 **	0.588 **	(0.736)	
7. ISE	3.843	0.575	0.036	0.025	0.163 **	0.608 **	0.367 **	0.480 **	(0.728)

*Note: N* = 2613. Internal reliabilities (alpha coefficients) for the constructs are given in parentheses on the diagonal. PFV = perceived functional value; BSC = bridging social capital; SVU = short-form video use; ISE = internet self-efficacy. * *p* < 0.05. ** *p* < 0.01.

**Table 2:** Testing the mediation effect of bridging social capital on the relationship between perceived functional value and short-form video use.

Predictors	Model 1 (Bridging Social Capital)		Model 2 (Short-Form Video Use)	
	B	t	B	t
Age	0.004	3.236 **	0.000	0.0123
Gender	−0.086	−3.0531 **	0.049	2.860 **
Education	−0.075	−5.167 ***	0.005	0.586
PFV	0.630	23.402 ***	0.485	27.100 ***
BSC			0.326	27.594 ***
*R* ^2^	0.183		0.501	
*F*	145.667 ***		523.034 ***	

*Note: N* = 2613. Each column is a regression model that predicts the variable at the top of the column. PFV = perceived functional value; BSC = bridging social capital. ** *p* < 0.01. *** *p* < 0.001.

**Table 3:** Testing the moderated mediation effect of internet self-efficacy between relationship of perceived functional value and bridging social capital.

	Model 3 (Bridging Social Capital)	
Predictors	B	t
Age	0.004	3.022 **
Gender	−0.061	−2.196 *
Edu	−0.090	−6.307 ***
PFV	−0.317	−2.448 *
ISE	−0.529	−3.942 **
PFV × ISE	0.270	6.111 ***
*R* ^2^	0.219	
*F*	122.065 ***	

*Note: N* = 2613. Each column is a regression model that predicts the variable at the top of the column. PFV = perceived functional value; ISE = internet self-efficacy. * *p* < 0.05. ** *p* < 0.01. *** *p* < 0.001.

## Data Availability

The original contributions presented in the study are included in the article, further inquiries can be directed to the corresponding author.
